# *Porphyromonas gingivalis* triggers microglia activation and neurodegenerative processes through NOX4

**DOI:** 10.3389/fcimb.2024.1451683

**Published:** 2024-10-14

**Authors:** Anna Magnusson, Rongrong Wu, Isak Demirel

**Affiliations:** ^1^ School of Medical Sciences, Örebro University, Örebro, Sweden; ^2^ Department of Periodontology and Implantology, Postgraduate Dental Education Center and School of Medical Sciences, Faculty of Medicine and Health, Orebro University, Örebro, Sweden

**Keywords:** microglia, Alzheimer’s disease, *Porphyromonas gingivalis*, neuroinflammation, NOX4

## Abstract

Periodontitis and infections with periodontal bacteria have been highlighted as risk factors for dementia. In recent years, attention has been drawn to the role of microglia cells in neurodegenerative diseases. However, there is limited knowledge of the influence of periodontal bacteria on microglia cells. The aim of the present study was to investigate the interactions between the periodontal bacteria *Porphyromonas gingivalis* and microglia cells and to unravel whether these interactions could contribute to the pathology of Alzheimer’s disease. We found, through microarray analysis, that stimulation of microglia cells with *P. gingivalis* resulted in the upregulation of several Alzheimer’s disease-associated genes, including NOX4. We also showed that *P. gingivalis* lipopolysaccharides (LPS) mediated reactive oxygen species (ROS) production and interleukin 6 (IL-6) and interleukin 8 (IL-8) induction via NOX4 in microglia. The viability of neurons was shown to be reduced by conditioned media from microglia cells stimulated with *P. gingivalis* LPS and the reduction was NOX4 dependent. The levels of total and phosphorylated tau in neurons were increased by conditioned media from microglia cells stimulated with *P. gingivalis* or LPS. This increase was NOX4-dependent. In summary, our findings provide us with a potential mechanistic explanation of how the periodontal pathogen *P. gingivalis* could trigger or exacerbate AD pathogenesis.

## 1 Introduction

Dementia has become a leading cause of disability and dependency worldwide due to the increasing elderly population. Alzheimer’s disease (AD) is the most common form of dementia and is associated with the extracellular assembly of the peptide amyloid-beta (Aβ) into plaques, as well as the intracellular deposition of phosphorylated tau neurofibrillary tangles (p-TauNFTs) in the brain. These abnormal protein structures contribute to neuronal cell death and have had a paramount role in the search for curative medications and explanatory models for AD (Selkoe, 2001). Since dementia patients exhibit neuroinflammation and neuronal cell loss consistent with an ongoing infection (Wyss-Coray and Rogers, 2012), an infectious etiology for AD has been proposed. Periodontitis and infections with periodontal bacteria have been highlighted as risk factors for dementia and AD (Chen et al., 2017; Holmer et al., 2018; Dominy et al., 2019; Nadim et al., 2020).

Periodontitis is an oral disease characterized by bacteria-driven inflammation, subsequent degradation of the supporting tissues of the teeth, and eventually tooth loss (Papapanou et al., 2018). One of the keystone pathogens in adult periodontitis is *Porphyromonas gingivalis* (*P. gingivalis*) (Hajishengallis and Lamont, 2014). *P. gingivalis* is a gram-negative, anaerobic, and proteolytic bacterium closely linked to the development of periodontitis. In conjunction with other oral anaerobes, *P. gingivalis* has been shown to degrade immunoglobulins, inhibit the complement system, and produce toxic components such as lipopolysaccharides (LPS) and gingipains. These factors help sustain bacterial viability during bacteremia, which commonly arises from dental procedures, tooth brushing, or surgical treatments (Hajishengallis and Lamont, 2014). There is strong evidence that oral bacteria, such as *P. gingivalis*, spread from the oral cavity into the bloodstream and potentially cause or accelerate pathological processes at distant sites (Hajishengallis and Chavakis, 2021). Studies have shown an association between *P. gingivalis* IgG and later incidence of AD (Beydoun et al., 2020) and lower cognitive function in AD patients (Leblhuber et al., 2020). *P. gingivalis* DNA was also found postmortem in the brains and cerebrospinal fluid (CSF) of patients with AD (Dominy et al., 2019). *P. gingivalis* secretes arginine or lysine-specific cysteine proteases (gingipains) (RgpA, RgpB, and Kgp) which have been shown to mediate cellular toxicity (Stathopoulou et al., 2009), induce the formation of Aβ plaques in mice brains (Ilievski et al., 2018), and have been identified in the brains of patients with Alzheimer’s disease (Dominy et al., 2019). *P. gingivalis* LPS, which mediates inflammation, has been detected postmortem in the brains of patients with AD (Poole et al., 2013). *P. gingivalis* has two major forms of LPS, LPS-A and LPS-O, which are linked to bacterial virulence (Rangarajan et al., 2008). Both LPS forms are linked to lipid A. A-LPS is an anionic polysacharide while O-LPS is a conventional O-antigen polysacharide found in most Gram negative-bacteria (Olsen and Singhrao, 2018).

In recent years, attention has been drawn to the role of microglial cells in neurodegenerative diseases (Gao et al., 2023). Microglial cells act as immune cells in the brain. They encompass the first line of defense against invading pathogens and tissue damage and influence neuronal development and function (Sousa et al., 2017). Resting microglia can be activated by pathogen‐associated molecular pattern molecules (PAMPs) or by damage‐associated molecular pattern molecules (DAMPs) and adopt either a pro- or anti-inflammatory phenotype. The pro-inflammatory phenotype is crucial for protection against invading microorganisms, but persistent activation is linked to neuroinflammation and neurotoxicity (Merighi et al., 2022).

There are only a few experimental studies that have investigated the influence of *P. gingivalis* on human microglial cells. Liu et al (Liu et al., 2017). and Nonaka et al (Nonaka and Nakanishi, 2020). showed that *P. gingivalis* induces gingipain-dependent microglia migration. Liu and colleagues also showed increased levels of the pro-inflammatory cytokines interleukin 6 (IL-6), tumor necrosis factor-alpha (TNF-α), and inducible nitric oxide synthase (iNOS) upon incubation with *P. gingivalis.* Inhibition of both Rgp- and Kgp-gingipains did not eliminate the observed increase. This suggests that other virulence factors may play a role in the *P. gingivalis*-induced inflammatory response (Liu et al., 2017). Tran and colleagues showed increased levels of disease-associated microglia markers [triggering receptor expressed on myeloid cells 2 (TREM-2), cluster of differentiation 86 (CD-86), iNOS, and Nitric oxide (NO) and pro-inflammatory cytokines upon incubation with *P. gingivalis* conditioned media (Tran et al., 2021).

Oxidative stress (OS) is implicated in AD pathology through the effect of reactive oxygen species (ROS) on neurodegeneration as well as Aβ and Tau pathology (Liu et al., 2023). Nicotinamide adenine dinucleotide phosphate (NADPH) oxidases (NOXs) are the primary enzymes responsible for ROS production, and recent research has suggested NOXs as a promising therapeutic target in neurodegenerative disorders (Tarafdar and Pula, 2018; Vermot et al., 2021). Various stimuli could activate the NOX enzymes, including bacterial LPS (Bedard and Krause, 2007). It has been demonstrated that microglia express NOX4 and that NOX4-generated ROS increase IL-6 levels (Li et al., 2009), suggesting a major role of NOX4 in neuroinflammation. The aim of the study was first to investigate the interactions between *P. gingivalis* and microglia cells and, second, to unravel whether these interactions could contribute to Alzheimer’s disease pathology.

## 2 Materials and methods

### 2.1 Cell and bacterial culture

The immortalized human microglial cell line HMC3 (American Type Culture Collection (ATCC), Manassas, VA, USA) was cultured in Dulbecco´s Modified Eagle Medium (DMEM) with 10% fetal bovine serum (FBS), 1mM non-essential amino acids, and 2mM L-glutamine (all from Thermo Fisher Scientific, Waltham, MA, USA) at 37°C in 5% carbon dioxide (CO_2_) atmosphere. The culture medium was changed to DMEM without FBS 18 hours prior to and during the experiments.

The neuroblastoma cell line SH-SY5Y (ATCC), was cultured in DMEM with 10% FBS, 1mM non-essential amino acids, and 2mM L-glutamine (all from Thermo Fisher Scientific) at 37°C in 5% CO_2_ atmosphere. Twenty-four hours after plating, neuronal differentiation was induced for 7 days by changing the medium to DMEM with 1% FBS and by adding 10µM retinoic acid (RA, Thermo Fisher Scientific) on days 1, 4, and 7 and 50ng/ml brain-derived neurotrophic factor (BDNF, R&D Systems, Minneapolis, MN, USA) on days 4 and 7 as previously described (De Medeiros et al., 2019).

*P. gingivalis* wild-type strain W50, the lysine gingipain (Kgp) mutant K1A, and the arginine gingipain (Rgp) mutant E8, kindly provided by Professor M.A. Curtis (Molecular Pathogenesis Group, Queen Mary, University of London), were anaerobically cultured in tryptic soy broth (TSB) supplemented with yeast extract (1mg/ml), hemin (5µg/ml), and menadione (1µg/ml) at 85% nitrogen (N_2_), 5% CO_2_, and 10% hydrogen (H_2_) at 37°C in an anaerobic chamber (Concept 400-M Anaerobic Workstation; Ruskinn Technology Ltd., Leeds, UK). *P. gingivalis* was grown for 48 h, centrifuged for 10 min at 10,000g, washed, and resuspended in PBS.

### 2.2 Stimulation of microglial cells

HMC3 cells were stimulated with *P. gingivalis* wild-type strain W50, the arginine gingipain (Rgp) mutant E8 and the lysine (Kgp) mutant K1A (MOI 10 or 100), *P. gingivalis* LPS (1µg/ml, A-LPS and O-LPS, Invivogen, San Diego, CA, USA), or phorbol myristate acetate (PMA, Thermo Fisher Scientific) at 37°C and 5% CO_2_ for 6 to 24 hours, depending on the experimental setup. The HMC3 cells were also pre-incubated with DMSO (vehicle), NOX4 inhibitor GLX-351322 (10µM, Thermo Fisher Scientific), PKC inhibitor bisindolylmaleimide I (10 µM, Santa Cruz Biotechnology Inc., Heidelberg, Germany), P38 inhibitor SB203580 (10 µM, Santa Cruz Biotechnology Inc), ERK inhibitor PD98059 (10 µM, Santa Cruz Biotechnology Inc. Heidelberg), or NF-κB inhibitor BAY11-7082 (2 µM, Enzo Life Sciences, NY, USA) for 1 hour prior to stimulation with bacteria or LPS. Supernatants were collected and stored at -80°C until further analysis. All experiments were conducted with a minimum of three replicates and independent biological experiments.

HMC3 cells were stimulated with W50 or LPS, with or without pre-incubation with the NOX4 inhibitor, for 24 hours. Supernatants were collected and centrifuged at 10,000g for 5 min and then sterilely filtered through a Filtropur S syringe filter with a PES membrane and a pore size of 0.2 µm (Sarstedt, Germany). These supernatants are referred to in this article as the conditioned media. The conditioned media were stored at -80°C until further analysis. No bacterial growth was observed after plating on fastidious anaerobe agar. All experiments were conducted with a minimum of three replicates and independent biological experiments.

### 2.3 RNA isolation and microarray analysis

HMC3 cells were stimulated with *P. gingivalis* strains W50, E8, and K1A (MOI 10) for 24 hours. Total RNA isolation was performed using an E.Z.N.A. Total RNA Kit I (Omega, Bio-tek, GA, USA) according to the manufacturer’s instructions. The Agilent TapeStation 2200 platform (Agilent Technologies, Palo Alto, CA, USA) was used to evaluate RNA quality and integrity. The RNA integrity number (RIN) was 10 for all RNA samples. The RNA was converted to cDNA through reverse transcription and complementary RNA (cRNA) was then synthesized through *in vitro* transcription. Labeled cRNA was prepared using the Low Input Quick Amp WT Labeling Kit (Agilent) and hybridization of the labeled cRNA was done onto Agilent SurePrint G3 (v3) Human Gene Expression 8 × 60 k (Agilent Technologies) glass arrays in a G2545A hybridization oven (Agilent) and subsequently scanned with a G2505C array laser scanner (Agilent Technologies). Feature Extraction software (version 10.7.3.1, Agilent Technologies) was used for image analysis and data extraction, as previously described (Klarström Engström et al., 2019). Gene expression data are available in the Gene Expression Omnibus (GEO) database with the accession number (GSE274532). The experiments were conducted with four independent biological experiments.

### 2.4 Detection of intracellular ROS

After incubation with inhibitors as described above, the medium was removed from the wells, and the HMC3 cells were incubated with 10 µM 2,7-dichlorodihydrofluorescein diacetate (H_2_DCFDA) (Thermo Fisher Scientific) for 30 min at room temperature. The cells were then washed with PBS, resuspended in DMEM, and stimulated with *P. gingivalis* W50 (MOI 10 or 100) or LPS (1µg/ml). Fluorescence was measured in a microplate reader (Cytation 3, BioTek, Winooski, VT, USA) every third minute for 24 hours at the excitation and emission wavelengths of 485 and 535 nm, respectively. All experiments were conducted with a minimum of three replicates and independent biological experiments.

### 2.5 Western blot analysis

HMC3 cells were lysed in radioimmunoprecipitation assay (RIPA) buffer supplemented with a protease and phosphatase inhibitor cocktail (Thermo Fisher Scientific). The protein concentrations were assessed using the DC protein assay (Bio-Rad Laboratories, Hercules, CA, USA). Protein samples and Laemmli buffer were mixed and boiled at 95°C for 10 min. The samples (10–20 µg of protein) were separated with 4%–15% SDS-polyacrylamide gel electrophoresis and then transferred to a polyvinylidene fluoride (PVDF) membrane (Bio-Rad Laboratories). Furthermore, 3% BSA was used to block the PVDF membrane for 1 h at room temperature. NOX4 was detected using a rabbit polyclonal antibody (Bio-Techne Ltd, UK). Phospho-PKC (p-PKC) was detected using a rabbit monoclonal antibody (Cell Signaling Technologies, Danvers, MA, USA). Phospho-38 (p38) was detected using a rabbit monoclonal antibody (Cell Signaling Technologies). Phospho-ERK (p-ERK) was detected using a mouse monoclonal antibody (Cell Signaling Technologies). PKC was detected using a mouse monoclonal antibody (Santa Cruz Biotechnology Inc., Heidelberg, Germany). p38 was detected using a rabbit polyclonal antibody (Cell Signaling Technologies). ERK was detected using a rabbit monoclonal antibody (Cell Signaling Technologies) and GAPDH was detected using a rabbit polyclonal antibody (Santa Cruz Biotechnology Inc). All the primary antibodies were incubated overnight at 4°C. As secondary antibodies, goat anti-mouse IgG (horseradish peroxidase, HRP) (Santa Cruz Biotechnology) and goat anti-rabbit IgG (HRP) (Santa Cruz Biotechnology) were used and incubated for 1 hour at room temperature. Luminata Forte Western HRP Substrate (Merck Millipore, Burlington, MA, USA) was used to develop the blots.

### 2.6 Cytokine release

HMC3 cells were pre-incubated with the NOX4 inhibitor for 1 hour prior to stimulation with *P. gingivalis* W50 (MOI 100), LPS (1µg/ml), or vehicle control DMSO for 24 hours at 37°C in 5% CO_2_ atmosphere. After centrifugation at 5,000g for 5 minutes, the supernatants were stored at -80°C until further analysis. An enzyme-linked immunosorbent assay (ELISA) was performed to measure the release of IL-6 and interleukin 8 (IL-8) from the HMC3 cells (ELISA MAX Deluxe Sets, BioLegend, San Diego, CA, USA) according to the manufacturer’s instructions. v All experiments were conducted with a minimum of three replicates and independent biological experiments.

### 2.7 Cell viability of neurons

The SH-SY5Y cells were stimulated with conditioned media (100 µl/well) from HMC3 cells for 24 and 48 hours. Cell viability was assessed by adding resazurin (44µM, Santa Cruz Biotechnology Inc) and incubating for an additional 4 hours at 37°C. Fluorescence was measured in a microplate reader (Cytation 3) at the excitation and emission wavelengths of 560 and 590 nm, respectively. All experiments were conducted with a minimum of three replicates and independent biological experiments.

### 2.8 Detection of total and phosphorylated tau

The SH-SY5Y cells were stimulated with conditioned media (250 µl/well) from HMC3 cells for 24 and 48 hours. The supernatants were collected, and 50 µl radioimmunoprecipitation assay (RIPA) buffer was used to lyse the remaining cells. The cell lysate and supernatants were mixed and centrifuged at 10,000g for 5 min and the supernatant were stored at -80°C until further analysis. Total and phosphorylated tau was analyzed using the Phospho(Thr231)/Total Tau Kit (Mesoscale Diagnostics, Rockville, MD, USA) according to the manufacturer’s instructions, and the plate was read using the QuickPlex SQ120 instrument (Meso Scale Discovery). All experiments were conducted with a minimum of three replicates and independent biological experiments.

### 2.9 Data analysis

All data shown are expressed as mean ± SEM. The differences between the groups were analyzed using unpaired Student’s t-test or one‐way ANOVA followed by the Bonferroni multiple testing correction. The statistical significance of the differences was considered at p < 0.05. The microarray analysis was performed using Gene Spring GX version 14.9 (Agilent Technologies) after per chip and gene 75th percentile shift normalization of the samples (n=4). For analyzing differences between the groups, one-way analysis of variance (ANOVA) was used. Significant gene entities (q < 0.05) were obtained using Tukey’s HSD *post hoc* test followed by the Bonferroni multiple testing correction and a fold change cut-off of ≥ 2. Disease ontology enrichment was done with DisGeNET (v7.0) and the significance was set at a p < 0.05.

## 3 Results

### 3.1 Microarray and gene-associations

**Figure 1:**
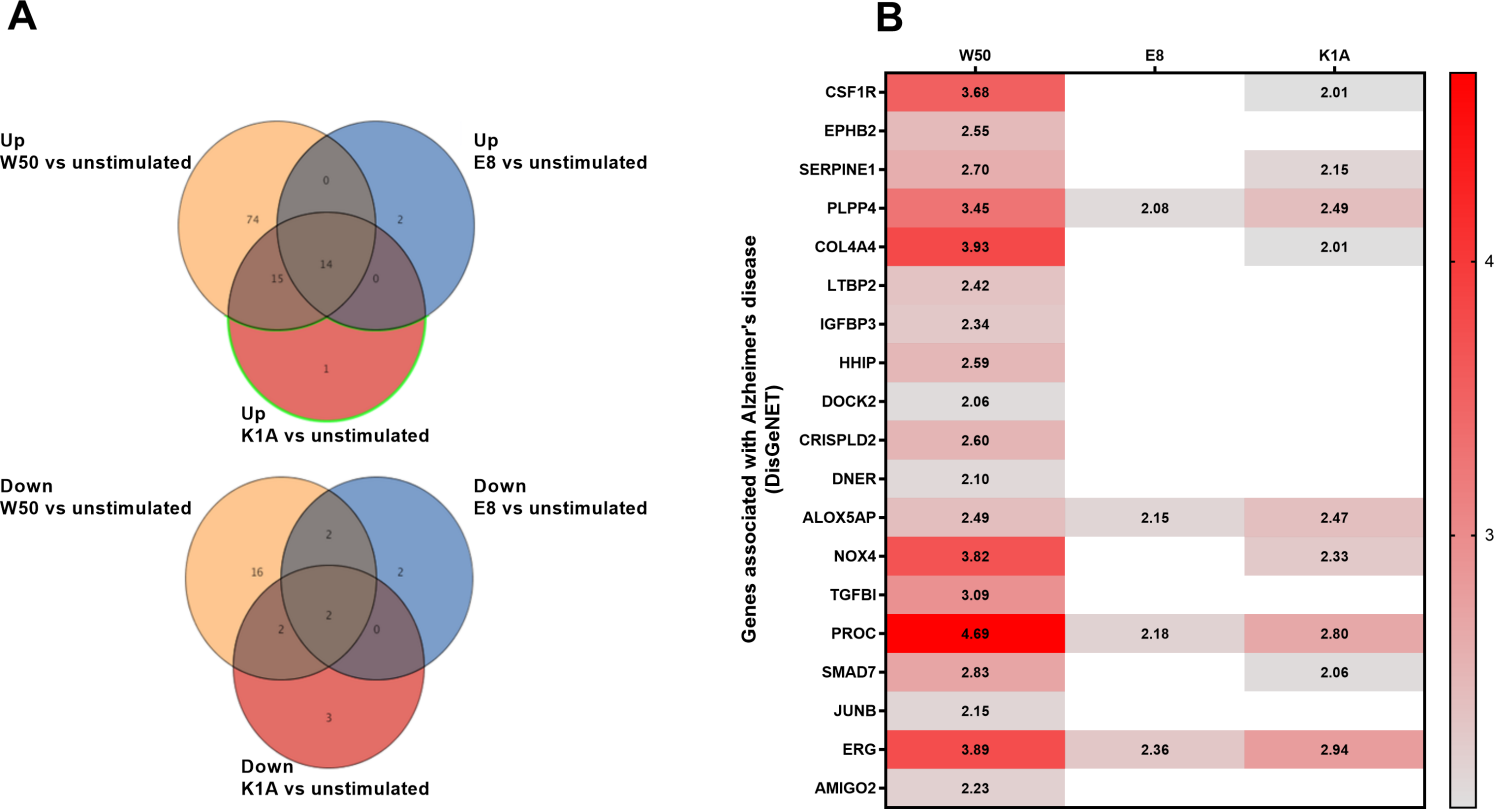
Microglia cells were infected with wild-type W50, the lysine gingipain (Kgp) mutant K1A, and the arginine gingipain (Rgp) mutant E8 for 24 hours followed by a microarray analysis. The Venn diagram illustrates the number of differentially expressed gene entities that were upregulated or downregulated after W50 (yellow), E8 (blue), or K1A (red) infection of microglia cells compared with unstimulated cells **(A)**. The heat map shows significantly (Fold change, p<0.05) altered gene entities associated with Alzheimer’s disease after W50, K1A, or E8 infection for 24 hours compared with unstimulated cells **(B)**. Data are presented as fold change from four independent experiments.

A whole genome microarray was conducted on the total RNA from the microglia cells stimulated with *P. gingivalis* wild-type strain W50 and the gingipain mutants E8 (Rgp) and K1A (Kgp). The analysis revealed 103 significantly upregulated (**Figure 1A**; **Supplementary Table S1**) and 22 downregulated (**Figure 1A**; **Supplementary Table S2**) genes after stimulation with W50 in comparison with the unstimulated microglia cells after 24 hours. Stimulation with E8 resulted in 16 upregulated (**Figure 1A**; **Supplementary Table S3**) and 6 downregulated genes (**Figure 1A**; **Supplementary Table S4**). Stimulation with K1A resulted in 30 upregulated (**Figure 1A**; **Supplementary Table S5**) and 7 downregulated genes (**Figure 1A**; **Supplementary Table S6**). DisgeNET disease enrichment analysis (**Figure 1B**) disclosed several upregulated genes associated with Alzheimer’s disease (CSF1R, EPHB2, SERPINE1, PLPP4, COL4A4, LTBP2, IGFBP3, HHIP, DOCK2, CRISPLD2, DNER, ALOX5AP, NOX4, TGFBI, PROC, SMAD7, JUNB, ERG, and AMIGO2). Out of the genes associated with Alzheimer’s disease, NOX4, a primary enzyme responsible for the production of ROS, was found to be of special interest for further evaluation.

### 3.2 *P. gingivalis* induces ROS production in microglia cells

**Figure 2:**
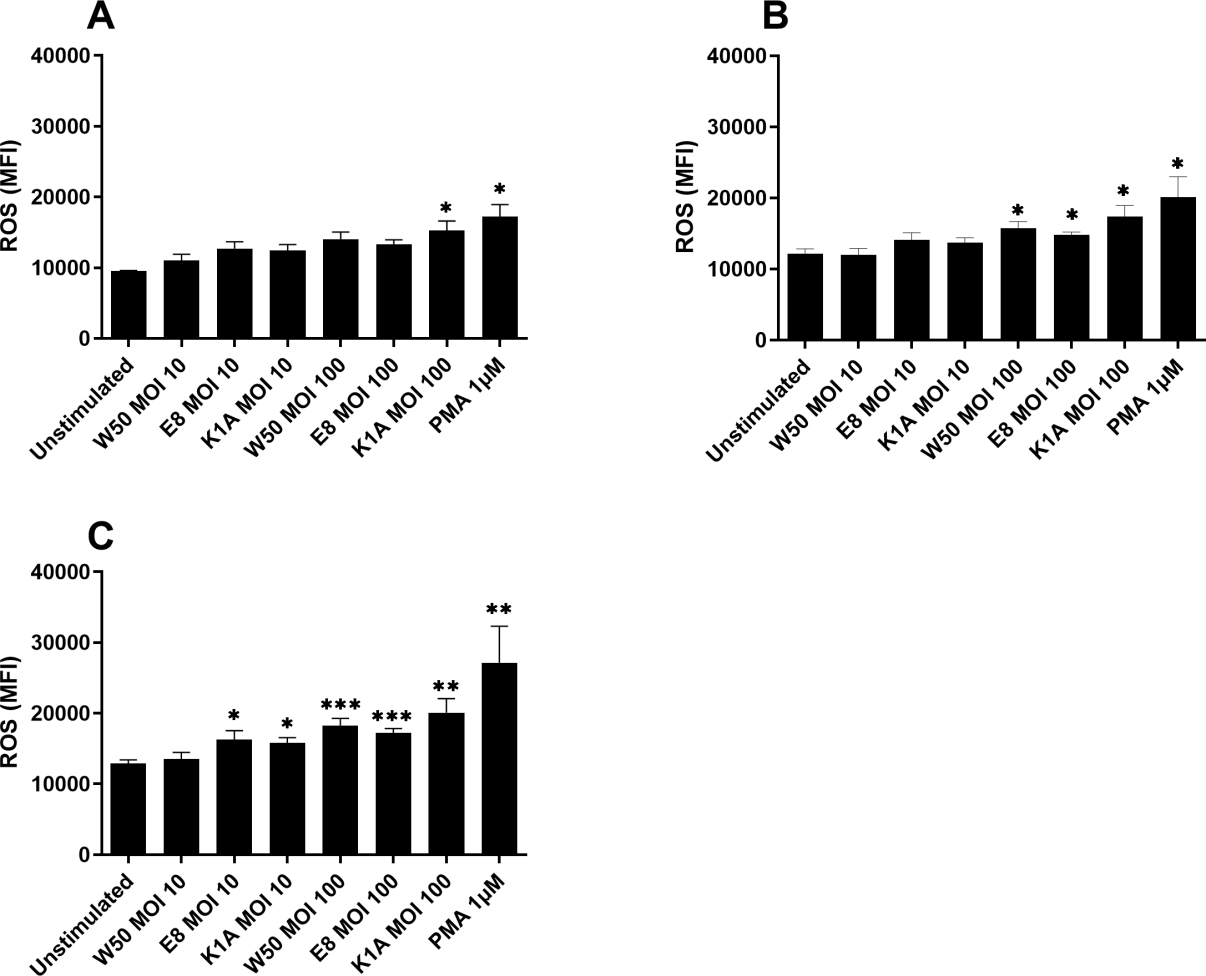
Microglia cells were infected with W50, the lysine gingipain (Kgp) mutant K1A, and the arginine gingipain (Rgp) mutant E8 for 6 **(A)**, 12 **(B)**, and 24 **(C)** hours and ROS production was evaluated. ROS production is presented as the mean fluorescence intensity (MFI). Data are presented as mean ± SEM of n=6 independent experiments. The asterisks indicate statistical significance: *p<0.05, **p<0.01 and ***p<0.001 vs. unstimulated.

We proceeded by investigating the production of ROS in microglia cells after stimulation with *P. gingivalis* W50, and the gingipain mutants E8 and K1A. After 6 hours, only K1A at MOI 100 and PMA demonstrated significantly upregulated ROS production in comparison with unstimulated cells (**Figure 2A**). After 12 hours, the ROS production was significantly increased by PMA, W50, E8, and K1A at MOI 100 (**Figure 2B**). After 24 hours, we observed that W50, E8, K1A at MOI 100, and E8, K1A at MOI 10 significantly increased ROS production compared with the unstimulated cells (**Figure 2C**).

### 3.3 LPS mediated ROS production via NOX4

**Figure 3:**
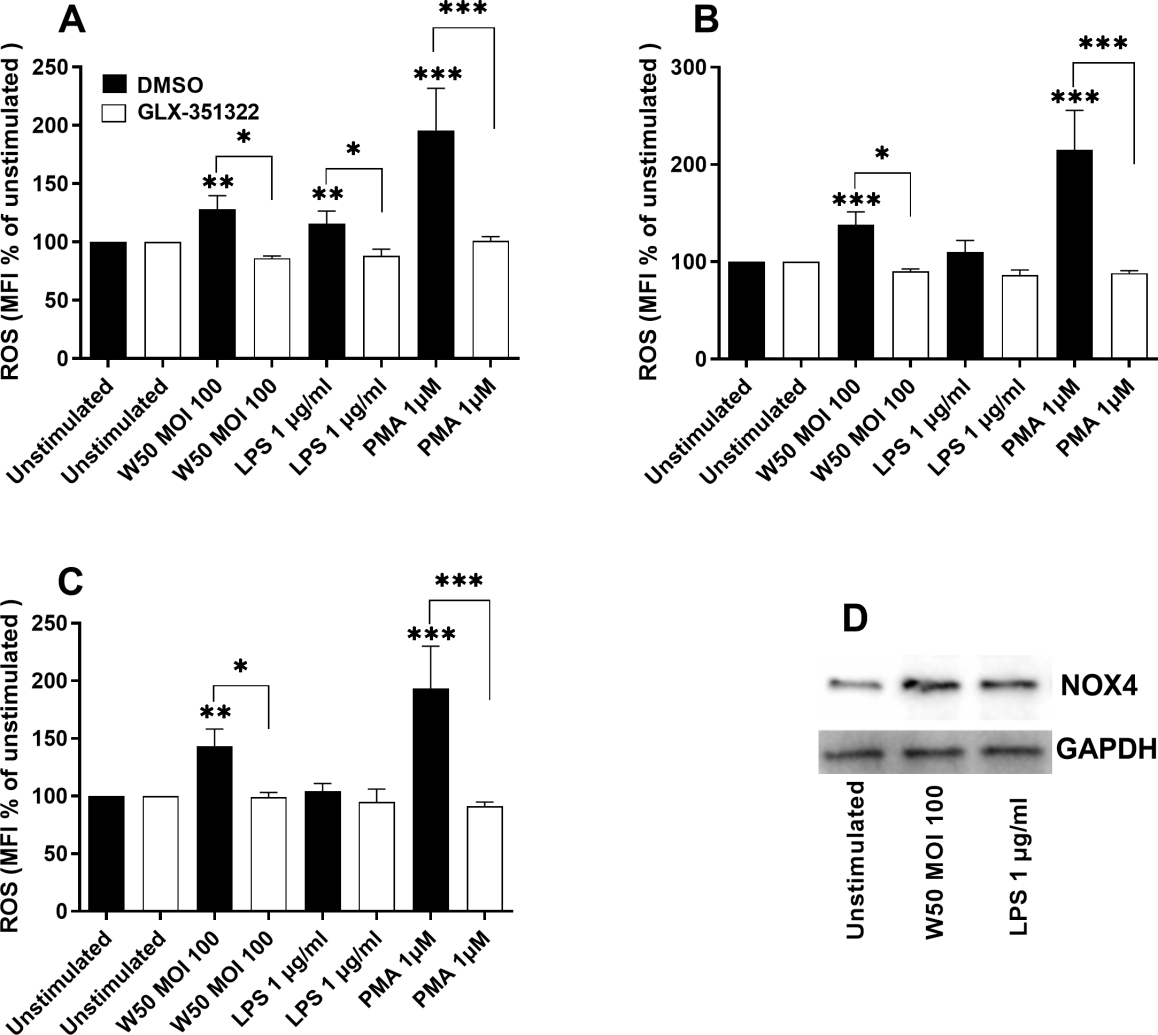
Microglia cells were pre-incubated with DMSO (vehicle) or NOX4 inhibitor GLX-351322 (10µM) for 1 hour prior to stimulation with W50, LPS, and PMA for 6 **(A)**, 12 **(B)**, and 24 **(C)** hours, and ROS production was evaluated. ROS production is presented as the percentage mean fluorescence intensity (MFI) vs the unstimulated cells. Western blot analysis was conducted to identify differences in the protein levels of NOX4 after W50 and LPS stimulation for 24 hours **(D)**. GAPDH was used as a loading control. Data are presented as mean ± SEM of n=3-4 independent experiments. The asterisks indicate statistical significance: *p<0.05, **p<0.01 and ***p<0.001 vs. unstimulated.

Since the gingipains appeared to be insignificant for ROS production, we continued by evaluating the role of *P. gingivalis* LPS. We found that both W50 and LPS caused an increased protein expression of NOX4 after 24 hours compared with unstimulated cells (**Figure 3D**). The ROS production was significantly increased by LPS after 6 hours (**Figure 3A**), but not after 12 (**Figure 3B**) and 24 hours (**Figure 3C**). We then wanted to investigate the role of NOX4 in the observed ROS production in microglia cells. Inhibiting NOX4 (GLX-351322) suppressed W50, LPS, and PMA mediated ROS production significantly after 6 (**Figure 3A**), 12 (**Figure 3B**), and 24 hours (**Figure 3C**).

### 3.4 *P. gingivalis* induces ROS via PKC, P38, and ERK signaling

**Figure 4:**
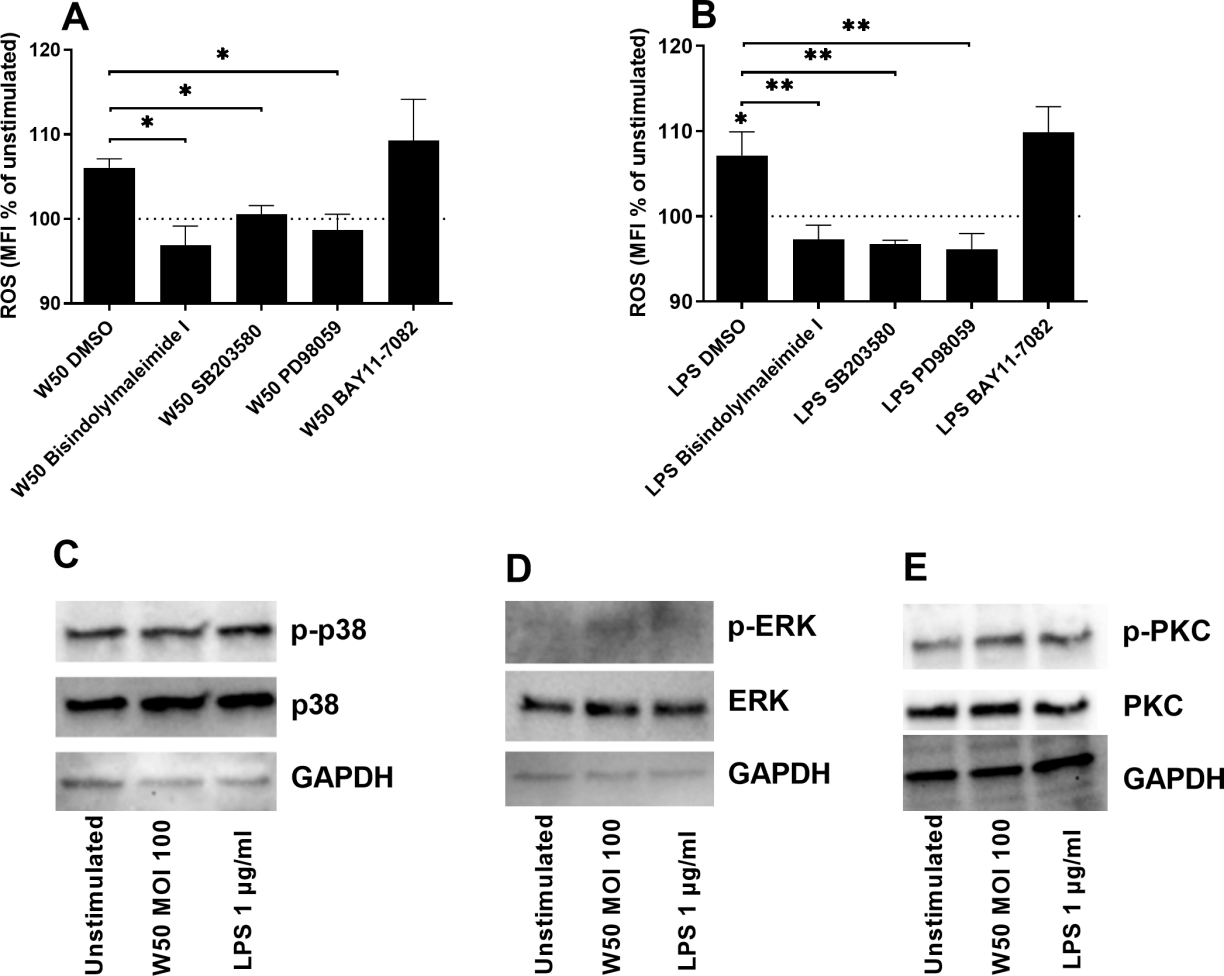
Microglia cells were pre-incubated with DMSO (vehicle), PKC inhibitor bisindolylmaleimide I (10 µM), P38 inhibitor SB203580 (10 µM), ERK inhibitor PD98059 (10 µM), or NF-κB inhibitor BAY11-7082 (2 µM) for 1 hour prior to stimulation with W50 MOI100 **(A)**, or LPS 1 µg/ml **(B)** for 24 hours and ROS was evaluated. ROS production is presented as the percentage mean fluorescence intensity (MFI) vs the unstimulated cells. The dotted line represents unstimulated cells. Western blot analysis was conducted to identify differences in the protein levels of p-p38/p38 **(C)**, p-ERK/ERK **(D)**, and p-PKC/PKC **(E)** after W50 or LPS stimulation for 3 **(D)**, 5 **(C)**, or 15 min **(E)**. GAPDH was used as a loading control. Data are presented as mean ± SEM of n=3 independent experiments. The asterisks indicate statistical significance: *p<0.05 and **p<0.01 vs. unstimulated.

The next step was to elucidate which signaling pathways mediate *P. gingivalis*-induced ROS production. We found that the inhibition of protein kinase C (PKC, bisindolylmaleimide I), p38 mitogen-activated protein kinase (p38, SB203580), and extracellular signal-related kinases (ERK, PD98059) but not the nuclear factor kappa-light-chain-enhancer of activated B cells (NFκB, BAY11-7082) significantly suppressed W50 (**Figure 4A**) or LPS (**Figure 4B**)-mediated ROS production after 24 hours. Western blot analysis showed that microglia cells exposed to W50 or LPS expressed higher levels of p-p38 (**Figure 4C**), p-ERK (**Figure 4D**), and p-PKC (**Figure 4E**) in comparison with unstimulated cells.

### 3.5 LPS-mediated induction of IL-6 and IL-8 is NOX4 dependent

**Figure 5:**
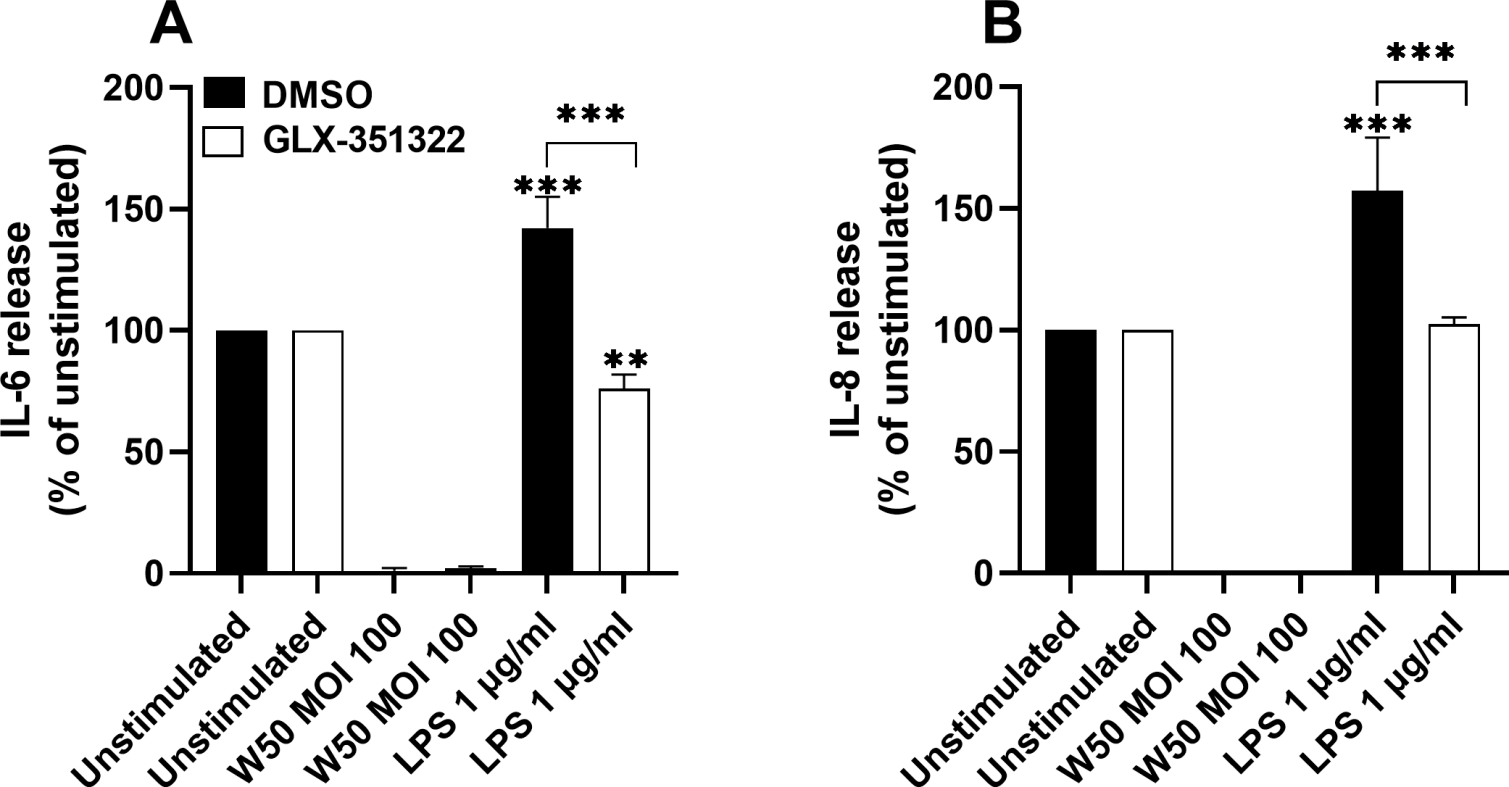
Microglia cells were pre-incubated with DMSO (vehicle) or NOX4 inhibitor GLX-351322 (10µM) for 1 hour prior to stimulation with W50 or LPS for 24 hours and IL-6 **(A)** or IL-8 **(B)** release was evaluated. Cytokine release is presented as the percentage vs the unstimulated cells. Data are presented as mean ± SEM of n=4 independent experiments. The asterisks indicate statistical significance: **p<0.01 and ****p<0.0001.

The next step was to evaluate the role of NOX4 in *P. gingivalis*-mediated neuroinflammation. Stimulation of microglia cells with *P. gingivalis* LPS resulted in significantly increased release of the pro-inflammatory cytokines IL-6 (**Figure 5A**) and IL-8 (**Figure 5B**) after 24 hours. Inhibition of NOX4 suppressed LPS-mediated IL-6 and IL-8 release (**Figure 5**). In addition, stimulation with *P. gingivalis* W50 did not result in detectable levels of IL-6 or IL-8. Furthermore, we did not observe any release of IL-1β or TNFα upon W50 or LPS stimulation after 24 hours (data not shown).

### 3.6 *P. gingivalis* LPS stimulated microglia cells mediate reduced neuron viability

**Figure 6:**
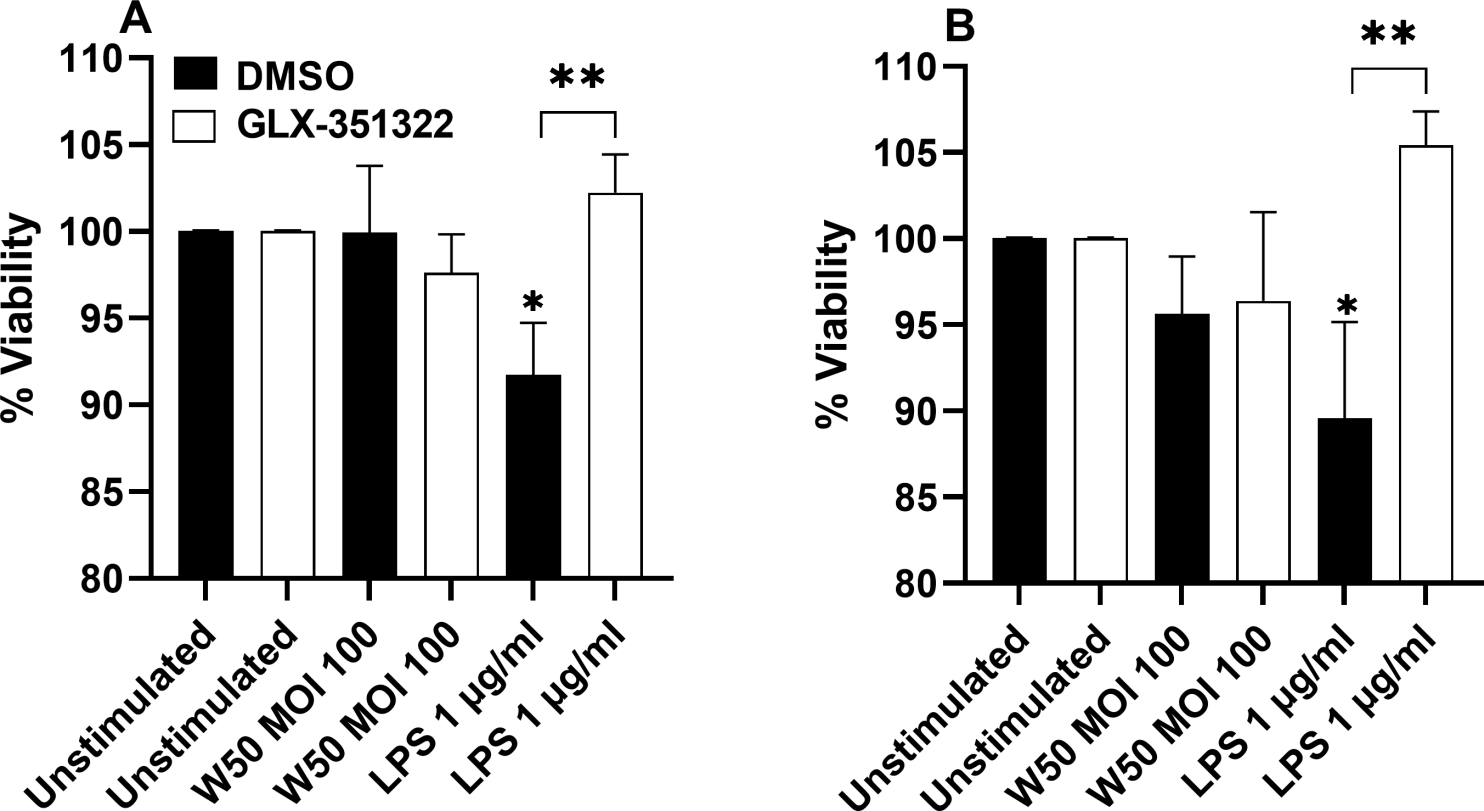
Neurons were exposed to conditioned media from microglia cells stimulated with W50, LPS, or from unstimulated cells, pre-incubation with DMSO (vehicle) or the NOX4 inhibitor GLX-351322 (10µM), and the neuron viability was then evaluated after 24 **(A)** or 48 hours **(B)**. Results are presented as the percentage viability vs the unstimulated cells. Data are presented as mean ± SEM of n=4 independent experiment. The asterisks indicate statistical significance: *p<0.05 and **p<0.01 vs. unstimulated.

Next, we wanted to explore the effect of the microglial cells on neuronal viability. Differentiated neuronal SH-SY5Y cells were stimulated with conditioned media from unstimulated microglia cells or microglia stimulated with W50 or LPS, in the presence or absence of the NOX4 inhibitor, for 24 and 48 hours. The neuron viability decreased upon stimulation with conditioned media from microglia cells stimulated with LPS in comparison with conditioned media from unstimulated cells after 24 (**Figure 6A**) and 48 hours (**Figure 6B**). In addition, conditioned media from LPS-stimulated microglia cells, pre-incubated with the NOX4 inhibitor, did not decrease neuron viability (**Figures 6A, B**). Conditioned media from W50-stimulated microglia cells induced a small but not significant reduction in neuron viability.

### 3.7 *P. gingivalis*-stimulated microglia cells mediate increased tau protein in neurons

**Figure 7:**
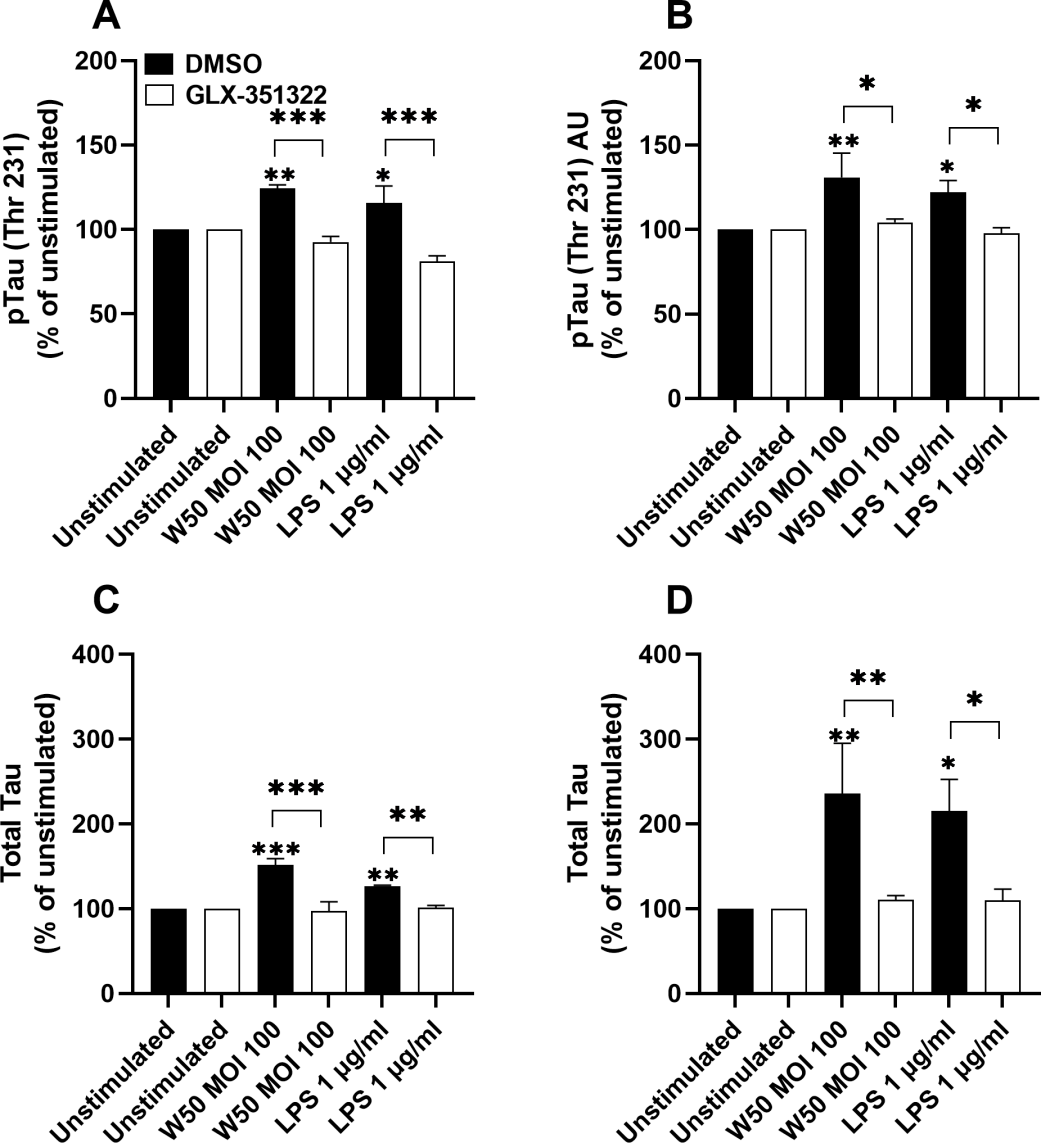
Neurons were exposed to conditioned media from microglia cells stimulated with W50, LPS, or from unstimulated cells, pre-incubation with DMSO (vehicle) or the NOX4 inhibitor GLX-351322 (10µM), and phosphorylated tau **(A, B)** or total tau **(C, D)** from neurons were evaluated after 24 **(A, C)** or 48 hours **(B, D)**. Results are presented as the percentage vs the unstimulated cells. Data are presented as mean ± SEM of n=4 independent experiment. The asterisks indicate statistical significance: *p<0.05, **p<0.01 and ***p<0.001 vs. unstimulated.

We proceeded to evaluate the ability of the conditioned media to increase the levels of total and phosphorylated tau in neurons. The amount of total and phosphorylated tau in neurons was increased when incubating neurons with conditioned media from microglia cells stimulated with *P gingivalis* W50 or LPS for 24 (**Figures 7A, C**) and 48 hours (**Figures 7B, D**). However, conditioned media from W50 and LPS-stimulated microglia cells, pre-incubated with the NOX4 inhibitor, failed to increase total and phosphorylated tau (**Figure 7**).

## 4 Discussion

In this study, we wanted to investigate the interaction between *P. gingivalis* and microglial cells and elucidate whether these interactions may contribute to Alzheimer’s disease pathology. We started by evaluating the global gene expression of microglia cells stimulated with *P. gingivalis* wild-type strain W50 and the gingipain mutants E8 (Rgp) and K1A (Kgp). The whole genome microarray analysis showed that several Alzheimer´s disease-associated genes were significantly upregulated in *P. gingivalis-*infected microglial cells. We found that the E8 mutant significantly upregulated only 4 of 19, and the K1A mutant 9 of 19 Alzheimer´s disease-associated genes in comparison to the wild-type strain W50. Furthermore, all the genes that were significantly upregulated by the mutants still had a lower gene expression compared to W50. Gingipains have previously been shown to mediate cellular toxicity (Stathopoulou et al., 2009) and induce the formation of Aβ plaques in mice brains (Ilievski et al., 2018). Gingipains have also been identified in the brains of patients with Alzheimer’s disease (Dominy et al., 2019) and they have been shown to mediate microglia migration via PAR2 activation (Liu et al., 2017; Nonaka and Nakanishi, 2020). Taken together, our findings indicate that gingipains could play an important role in the interaction between *P. gingivalis* and microglia cells.

When evaluating the genes associated with Alzheimer’s disease, NOX4, which was upregulated, was found to be of particular interest. NOX4 has been linked to oxidative stress and tauopathies in neurodegenerative diseases (Tarafdar and Pula, 2018; Luengo et al., 2022). NOX inhibitors have also been suggested as a potential therapeutic approach in neurodegenerative diseases (Barua et al., 2019; Vermot et al., 2021). Interestingly, the E8 mutant did not increase the expression of NOX4 like the wild-type W50 and mutant K1A did. Next, we validated that *P. gingivalis* induced significantly increased ROS production in microglia cells. Interestingly, the increase was independent of gingipains. Instead, *P. gingivalis* LPS appeared to generate the same production of ROS as the bacteria itself, suggesting a key role of LPS in *P. gingivalis*-induced oxidative stress. We also validated that NOX4 was upregulated by W50 and LPS at the protein level. Zhan and colleagues (Zhan et al., 2018) have previously summarized evidence of LPS acting as a trigger of neuroinflammation and AD. They highlighted that LPS activates leukocyte and microglial TLR4-CD14/TLR2 receptors, leading to NFκB-mediated cytokine production. This increase in cytokines leads to elevated Aβ levels, damages oligodendrocytes, and causes myelin injury observed in the brains of patients with AD. Additionally, since Aβ1–42 is an agonist for TLR4, this could create a cycle contributing to the progression of AD. In order to link the observed ROS increase to NOX4, a NOX4 inhibitor was used. We found that both W50 and LPS-mediated ROS production was NOX4-dependent. These results are in agreement with Li and colleagues (Li et al., 2009), who showed that ROS production in human microglia cells is NOX4 dependent, and with Meng and colleagues, who showed LPS activation of NOX in macrophages (Meng et al., 2013). Taken together, we have shown that *P. gingivalis*-mediated ROS production in microglia cells is mediated by LPS through NOX4.

Next, we proceeded by evaluating which signaling pathways mediate *P. gingivalis*-induced ROS production. We found that W50 and LPS induced ROS production via p38, ERK, and PKC, but not NF-κB. It has previously been shown that ERK, p38, and PKC can regulate NOX2, NOX4 activation, and ROS production in several cell types, including human microglia cells. These pathways are also strongly associated with neuroinflammatory responses in microglia cells (Haslund-Vinding et al., 2017; Zhang et al., 2018; Kim et al., 2020; Simpson and Oliver, 2020; Xun et al., 2022). Taken together, our findings indicate that p38, PKC, and ERK, but not NF-κB signaling, mediates *P. gingivalis-*induced ROS production in microglia cells.

We also investigated the role of NOX4 in *P. gingivalis*-mediated release of pro-inflammatory cytokines and chemokines from microglia cells. We found that *P. gingivalis* LPS, but not the bacteria, induced a NOX4-dependent increase in IL-6 and IL-8, but not IL-1β and TNFα, released from microglia cells. The cytokines could not be detected after *P. gingivalis* stimulation due to the protease activity of the gingipains, which degrades them (Jayaprakash et al., 2014). Both IL-6 and IL-8 are pro-inflammatory cytokines involved in different neurodegenerative disorders including AD (Shen et al., 2019). Increased levels of IL-6 have been associated with both the accumulation of amyloid beta plaques and neurofibrillary tangles, as well as blood-brain barrier dysfunction (Shan et al., 2024). Our results are in agreement with Li and colleagues who showed that ROS mediated by NOX4 regulated IL-6 levels in microglia cells (Li et al., 2009). A recently published study by Verma and colleagues (Verma et al., 2024) showed, in contrast to our results, that *P. gingivalis* LPS induced increased IL-1β and TNFα levels in microglia cells. This might be due to differences in stimulation time and LPS concentration between our studies. Verma and colleagues found the highest release of cytokines after 4 hours and they also used 10 µg/ml LPS. Our experiments were conducted with 1 µg/ml LPS for 24 hours. Earlier studies on mouse microglial cell lines have also shown upregulation of IL-1β and TNFα (Qiu et al., 2021; Inoue et al., 2024). Hence, the NOX4-dependent increase in IL-6 and IL-8 in human microglia cells suggests a neuroinflammatory role of *P. gingivalis* LPS.

The next step was to investigate how the *P. gingivalis*-mediated effects on microglial cells influenced neuronal viability and total and phosphorylated tau levels, two neuropathological hallmarks of Alzheimer’s disease. We showed that the viability of neurons decreased upon stimulation with conditioned media from microglia cells stimulated with *P. gingivalis* LPS. We also showed that this reduction in viability was NOX4-dependent. These findings confirm the potential neurotoxic effect of activated microglial cells that has been recently highlighted (Gao et al., 2023). We also showed that total and phosphorylated tau levels were elevated when incubating neurons with conditioned media from W50 or LPS-treated microglial cells. Similar to the cell viability, this increase was NOX4-dependent. Tran and colleagues (Tran et al., 2021) also found significantly lower neuronal viability and higher levels of phosphorylated tau when neurons were incubated with conditioned media from *P. gingivalis*-treated microglial cells compared with conditioned media from bacteria only. This indicates that the microglia-mediated neuroinflammation, triggered by *P. gingivalis* and LPS, may be neurodegenerative and driven by NOX4.

This study provides insight into the interaction between the oral pathogen *P. gingivalis*, microglia, and neuron cells. The results of this study give us a potential mechanistic explanation of how periodontitis, with systemic spread of bacteria such as *P. gingivalis*, could trigger or exacerbate AD pathogenesis. Hence, our findings support the infection hypothesis of Alzheimer’s disease.

## Data Availability

The datasets presented in this study can be found in online repositories. The names of the repository/repositories and accession number(s) can be found in the article/**Supplementary Material**.
